# Effects of Folic Acid Supplementation on Inflammatory Markers: A Grade-Assessed Systematic Review and Dose–Response Meta-Analysis of Randomized Controlled Trials

**DOI:** 10.3390/nu13072327

**Published:** 2021-07-06

**Authors:** Omid Asbaghi, Damoon Ashtary-Larky, Reza Bagheri, Seyedeh Parisa Moosavian, Behzad Nazarian, Reza Afrisham, Mahnaz Rezaei Kelishadi, Alexei Wong, Frédéric Dutheil, Katsuhiko Suzuki, Amirmansour Alavi Naeini

**Affiliations:** 1 Cancer Research Center, Shahid Beheshti University of Medical Sciences, Tehran 14167-53955, Iran; omid.asbaghi@gmail.com; 2 Nutrition and Metabolic Diseases Research Center, Ahvaz Jundishapur University of Medical Sciences, Ahvaz 61357-15794, Iran; damoon_ashtary@yahoo.com; 3 Department of Exercise Physiology, University of Isfahan, Isfahan 81746-73441, Iran; will.fivb@yahoo.com; 4 Department of Clinical Nutrition, School of Nutrition and Food Science, Isfahan University of Medical Sciences, Isfahan 81746-73461, Iran; p_moosavian@yahoo.com; 5 Student Research Committee, Lorestan University of Medical Sciences, Khorramabad 68138-33946, Iran; nazarianbehzad969@yahoo.com; 6 Department of Clinical Laboratory Sciences, Faculty of Allied Medicine, Tehran University of Medical Sciences, Tehran 14176-13151, Iran; rezaafrisham@yahoo.com; 7 Department of Community Nutrition, School of Nutrition and Food Science, Isfahan University of Medical Sciences, Isfahan 81746-73461, Iran; m.rezaei81@yahoo.com; 8 Department of Health and Human Performance, Marymount University, Arlington, VA 22207, USA; awong@marymount.edu; 9 CNRS, LaPSCo, Physiological and Psychosocial Stress, CHU Clermont-Ferrand, University Hospital of Clermont-Ferrand, Preventive and Occupational Medicine, Université Clermont Auvergne, WittyFit, F-63000 Clermont-Ferrand, France; fred_dutheil@yahoo.fr; 10 Faculty of Sport Sciences, Waseda University, 2-579-15 Mikajima, Tokorozawa 359-1192, Japan

**Keywords:** inflammation, folic acid, metabolic diseases

## Abstract

It has been theorized that folic acid supplementation improves inflammation. However, its proven effects on inflammatory markers are unclear as clinical studies on this topic have produced inconsistent results. To bridge this knowledge gap, this systematic review and meta-analysis of randomized controlled trials (RCTs) aimed to evaluate the effects of folic acid supplementation on serum concentrations of the inflammatory markers C-reactive protein (CRP), interleukin-6 (IL-6), and tumor necrosis factor-alpha (TNF-α). **Methods:** To identify eligible RCTs, a systematic search up to April 2021 was completed in PubMed/Medline, Scopus, Web of Science, EMBASE, Cochrane databases, and Google Scholar using relevant keywords. A fix or random-effects model was utilized to estimate the weighted mean difference (WMD) and 95% confidence interval (95% CI). **Results:** Twelve RCTs were included in the present meta-analysis. The pooled analysis revealed that serum concentrations of CRP (WMD: −0.59 mg/L, 95% CI −0.85 to −0.33, *p* < 0.001) were significantly reduced following folic acid supplementation compared to placebo, but did not affect serum concentrations of IL-6 (WMD: −0.12, 95% CI −0.95 to 0.72 pg/mL, *p* = 0.780) or TNF-α (WMD: −0.18, 95% CI −0.86 to 0.49 pg/mL, *p* = 0.594). The dose–response analysis demonstrated a significant relationship between an elevated dosage of folic acid supplementation and lower CRP concentrations (*p* = 0.002). **Conclusions:** We found that folic acid supplementation may improve inflammation by attenuating serum concentrations of CRP but without significant effects on IL-6 and TNF-α. Future RCTs including a larger number of participants and more diverse populations are needed to confirm and expand our findings.

## 1. Introduction

Inflammation is a protective reaction by an organism in response to injury, irritation, or infection, eliminating harmful stimuli and initiating the healing process [1]. Harmful agents activate inflammatory cells, such as neutrophils and macrophages, and induce the release of proinflammatory molecules, including C-reactive protein (CRP), tumor necrosis factor-alpha (TNF-α), and interleukin-6 (IL-6). Although inflammation is an essential component for a proper immune response and the maintenance of homeostasis in the body [2,3], chronic inflammation plays a key role in the onset and progression of several chronic diseases, including diabetes [4], cardiovascular diseases [5], neurodegenerative diseases [6], rheumatoid arthritis [7], and cancer [8]. Popular approaches suggest controlling inflammation through pharmacological [9,10,11] and dietary interventions [12,13]. Despite the considerable benefits of pharmacological therapies, these may exert undesirable side effects and may not be tolerated by certain individuals [14]. Therefore, it is essential to find nutraceuticals and natural compounds with anti-inflammatory effects that may serve as alternative therapies to pharmacological interventions.

Numerous studies have found the effective role of dietary supplements on human health [15,16,17]. Folate, a family of water-soluble B vitamins, is a generic term used for natural folates in food and folic acid, with the synthetic form available in fortified foods and supplements [18]. Folate is essential for one-carbon metabolism, which plays a role in various cellular reactions such as deoxyribonucleic acid (DNA) synthesis, repair, and methylation [19]. Mammals cannot synthesize folate de novo and depend entirely on absorption from the diet or supplements [18]. However, the bioavailability of natural food folates is approximately 50% of that of the synthetic form of folic acid [19].

Folate deficiency has been implicated in numerous adverse health conditions, including cardiovascular disease, cognitive dysfunction, neural tube defects, and cancer [20,21]. Recently, evidence from several clinical studies [22,23,24,25] focused on the beneficial effects of folic acid supplementation on inflammation; however, these studies yielded inconsistent results. Moreover, these studies used different sample sizes, study durations, and supplement dosages, which makes it challenging to draw a firm association between folic acid supplementation and inflammatory markers. Therefore, we performed the present systematic review and meta-analysis to establish the overall effects of folic acid supplementation on inflammatory markers.

## 2. Materials and Methods

The present systematic review and meta-analysis was performed in accordance with the Preferred Reporting Items for Systematic Reviews and Meta-Analyses (PRISMA) protocol [26].

### 2.1. Search Strategy

To find relevant prospective studies, we executed a systematic literature search in electronic databases including PubMed/Medline, Scopus, Web of Science, EMBASE, Cochrane databases, and Google Scholar from inception until April of 2021. The combination of MESH and non-MESH terms were used for the search, as follows: (“folate” OR “folic acid” OR “Vitamin M” OR “Vitamin B9” OR “Folacin” OR “Folvite” OR “Pteroylglutamic Acid” OR “folates” OR “tetrahydrofolates” OR “Formyltetrahydrofolates”) AND (“Inflammation” OR “inflammatory” OR “Tumor necrosis factor” TNF-α OR TNF OR “C-Reactive protein” OR “c reactive protein” OR “high-sensitivity CRP” OR hs-CRP OR CRP OR hsCRP OR hs-CRP OR “Cytokine” OR “Interleukin” OR “IL-6”) AND (Intervention OR “Intervention Study” OR “Intervention Studies” OR “controlled trial” OR randomized OR randomized OR random OR randomly OR placebo OR “clinical trial” OR Trial OR “randomized controlled trial” OR “randomized clinical trial” OR RCT OR blinded OR “double blind” OR “double blinded” OR trial OR “clinical trial” OR trials OR “Pragmatic Clinical Trial” OR “Cross-Over Studies” OR “Cross-Over” OR “Cross-Over Study” OR parallel OR “parallel study” OR “parallel trial”). There were no language restrictions. Moreover, the bibliographies of related studies were scrutinized to find potential missing studies.

### 2.2. Inclusion Criteria

We included studies that satisfied the following criteria: (1) randomized placebo-controlled trials (RCTs) with either parallel or crossover designs, (2) those carried out on adult cohorts (≥18 years), (3) examined the effects of folic acid supplementation on serum concentrations of CRP, IL-6, and TNF-α, and (4) RCTs that provided sufficient data (number of participants per intervention, means, and standard deviations (SDs)) for baseline and post-intervention outcome measures.

### 2.3. Exclusion Criteria

Studies were excluded when: (1) no placebo-controlled trial was utilized, (2) there was a lack of information for the outcomes in the intervention or control groups, (3) they were performed on children, pregnant women, or animals, (4) they were grey literature such as conference papers, dissertations, and patents, and (5) the effect of folic acid supplementation in combination with other supplements and exercise interventions was examined.

### 2.4. Data Extraction

Data were independently extracted from eligible studies by two researchers (OA and MR), and a chief investigator (DA) made a final assessment on any inconsistencies to reach a consensus. The following data were included: first author’s last name, year of publication, study location, study duration, type and dosage of folic acid supplements, mean age, body mass index (BMI), gender, study design, health status of participants, number of participants in each group, as well as the means ± SDs of the outcome measures in the intervention and control groups at baseline and post-intervention (or change values) for inflammatory markers. If any studies reported inadequate data for meta-analysis, authors were contacted through e-mail.

### 2.5. Quality Assessment

The risk of bias in qualified studies was assessed via the Cochrane scoring system [27]. Each included study was evaluated based on seven items: the random sequence generation, blinding of participants, investigator and outcome assessment, concealed allocation, selective reporting, incomplete outcome data, and other biases (Table 1).

### 2.6. Data Synthesis and Statistical Analysis

The meta-analysis was carried out using Stata, version 14 (StataCorp, College Station, TX, USA). Means and SDs of the outcome measures (CRP, IL-6, and TNF-α) reported for the intervention and control groups were used to obtain the overall estimates. If the SD of the mean difference was not reported in the studies, we calculated it using the following formula: SD change = square root ([SD baseline]^2^ + [SD final]^2^ − [2R × SD baseline × SD final]) [28]. Effect sizes for all variables were listed as weighted mean differences (WMDs) and 95% confidence intervals (CI) [29]. We calculated heterogeneity between study-specific estimates using the *Q*-test and the I^2^ index (I^2^ > 40% considered as considerable heterogeneity). A sensitivity analysis was carried out to determine the effect of each study on the overall effect size [30]. Publication bias was measured by the funnel plot inspection as well as the Egger’s test. Moreover, we performed a one-stage robust error meta-regression (REMR) model, which is based off inverse variance weighted least squares regression and cluster robust error variances for the dose–response analysis between folic acid supplementation and inflammatory markers [31]. The overall certainty of evidence across the studies was graded according to the Grading of Recommendations Assessment, Development, and Evaluation (GRADE) guidelines working group. The quality of evidence was classified into four categories, based off the corresponding evaluation criteria: high, moderate, low, and very low [32].

**Table 1:** Quality assessment.

Studies	Random Sequence Generation	Allocation Concealment	Selective Reporting	Other Sources of Bias	Blinding (Participants and Personnel)	Blinding (Outcome Assessment)	Incomplete Outcome Data	Overall Quality
Mangoni et al., 2003 [23]	L	H	H	H	H	H	L	Fair
Spoelstra-de Man et al., 2004 [33]	L	H	L	H	L	H	L	Good
Durga et al., 2005 [34]	L	H	H	H	L	H	L	Good
Klerk et al., 2005 [35]	L	H	H	H	L	H	L	Good
Olini et al., 2006	L	H	H	H	H	H	L	Fair
Title et al., 2006 [24]	L	H	H	H	L	L	L	Good
Moens et al., 2007 [36]	L	H	H	H	L	H	L	Good
Bahmani et al., 2014 [37]	L	H	H	H	L	H	L	Good
Asemi et al., 2016 [38]	L	H	H	H	L	H	L	Good
Chen et al., 2016 [39]	L	H	H	H	H	H	L	Fair
Talari et al., 2016 [22]	L	H	H	H	L	H	L	Good
Bahmani et al., 2018 [35]	L	H	H	H	L	H	L	Good

Abbreviations: L, low; H, high.

## 3. Results

### 3.1. Study Selection

**Figure 1:**
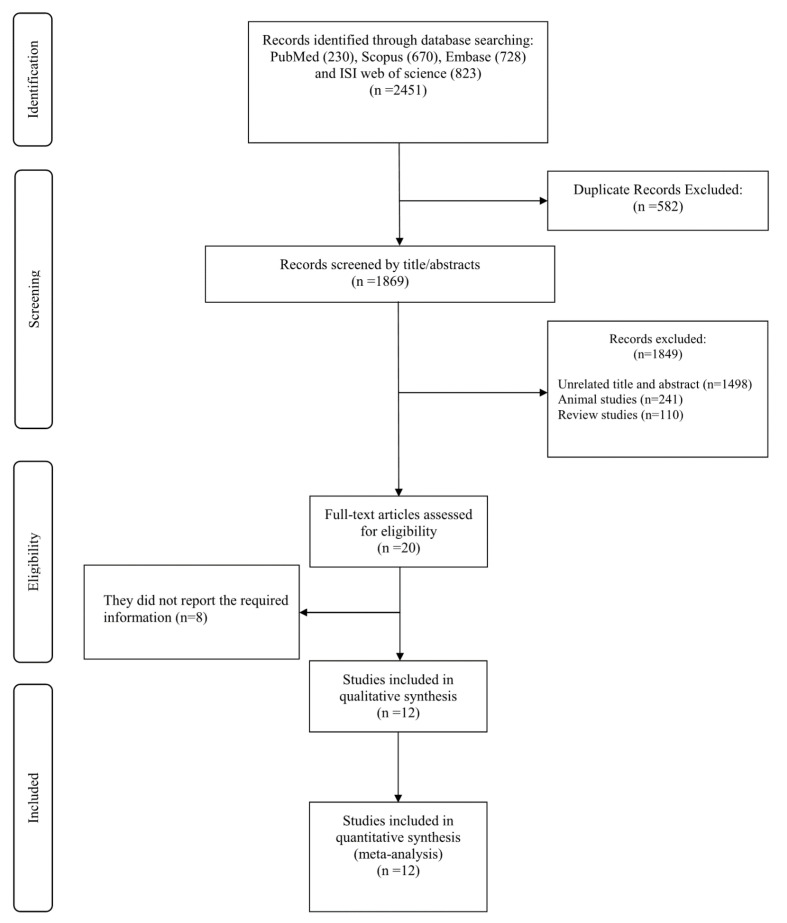
Flowchart of study selection for inclusion.

In our primary search, we identified a total of 2451 peer-reviewed studies. Among these, 582 studies were excluded due to duplication. Consequently, 1869 relevant studies remained for title and abstract evaluation, 1849 of which were excluded due to the following reasons: unrelated titles and abstracts (*n* = 1498), animal studies (*n* = 241), and review studies (*n* = 110). The remaining 20 full-text studies were assessed for eligibility and out of these, 8 studies were removed due to a lack of required information. Finally, 12 studies were included in the current study. The selection process is summarized in Figure 1.

### 3.2. Characteristics of the Included Studies

The features of the included studies are shown in Table 2. There were 12 studies with a total of 1392 participants. The studies were conducted between 2003 and 2018 and their sample size varied from 19 [24] to 530 [34] participants. The mean age of the participants ranged from 24.1 [37] to 68.1 [39] years old and the mean baseline BMI varied from 23.256 [39] to 30.7 [40] kg·m^−2^. Studies were conducted in The Netherlands [33,34,35], United Kingdom [23], Iran [22,37,38,40], Italy [41], Canada [24], Belgium [36], and China [39]. The supplementation period ranged from 2 [24] to 52 [34,35] weeks. The daily mentioned dosage of folic acid varied between 0.8 [34,35] and 10 [24,36] mg/d. Two studies used a crossover design [24,36], while the other ten investigations used a parallel-arm design. Among the included studies, three studies used only females [37,38,40], while other studies were performed on both sexes. Moreover, gender was not identified in one study [35]. Some studies included participants with diseases and complications such as polycystic ovary syndrome [35], type 2 diabetes mellitus [24,33], acute myocardial infarction [36], cervical intraepithelial neoplastic grade 1 [38], Alzheimer’s disease [39], metabolic syndrome [22], and endometrial hyperplasia [40], while other investigations used healthy smokers [23] as well as overweight [41] and older adults [34,35].

### 3.3. Quality Assessment

All studies had a low risk for random sequence generation and incomplete outcome data. Moreover, all studies had a high risk for allocation concealment and other sources of bias. In addition, all studies had a high risk of bias regarding selective reporting except for one [33]. Most studies had a low risk for blinding participants and personnel, with the exception of three studies [23,39,41]. Furthermore, all studies but one [24] had a high risk for blinding outcome assessment (Table 1).

### 3.4. The Effect of Folic Acid Supplementation on Serum Concentrations of CRP

**Figure 2:**
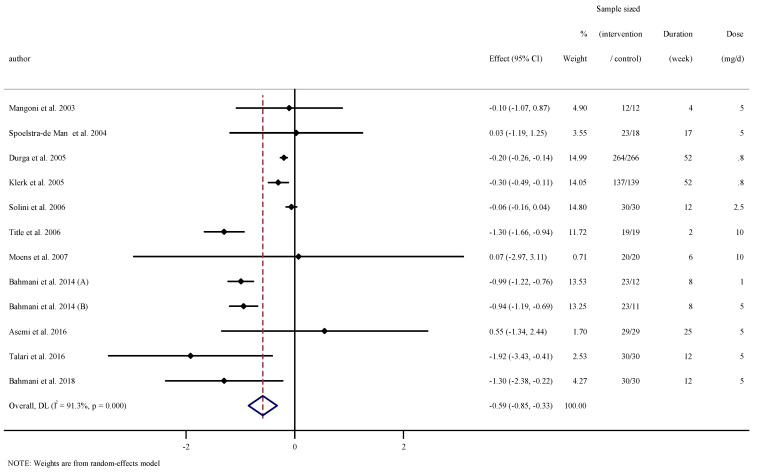
Forest plot detailing weighted mean difference and 95% confidence intervals (CIs) for the effect of folic acid supplementation on serum concentrations of CRP.

Combining 12 effect sizes from 11 RCTs [22,23,24,33,34,35,36,37,38,40,41], including a total sample size of 1279 participants, we found a significant effect of folic acid supplementation on serum concentrations of CRP (WMD: −0.59 mg/L, 95% CI −0.85 to −0.32, *p* < 0.001). Heterogeneity between studies was significant (I^2^ = 91.3%, *p* < 0.001, Figure 2). Subgroup analysis showed that baseline serum concentrations of CRP, duration of intervention, dosage, and the participants’ age and gender explained this heterogeneity. We observed that folic acid supplementation significantly reduced serum concentrations of CRP in all subgroups (Table 3).

### 3.5. The Effect of Folic Acid Supplementation on Serum Concentrations of IL-6

**Figure 3:**
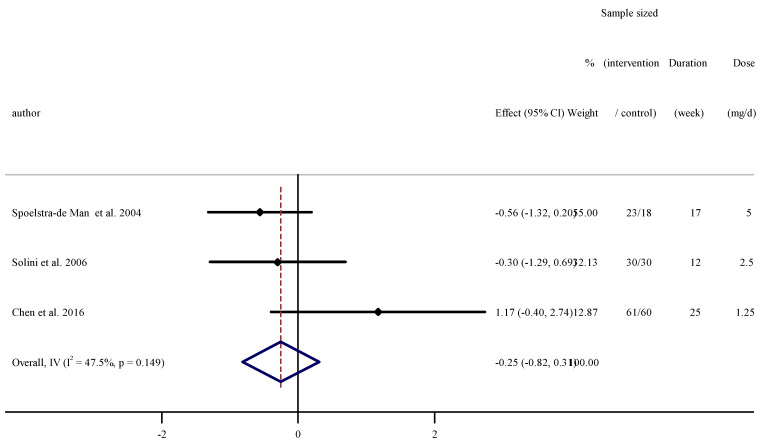
Forest plot detailing weighted mean difference and 95% confidence intervals (CIs) for the effect of folic acid supplementation on serum concentrations of IL-6.

Pooled effect sizes from three RCTs [33,39,41] and 222 participants were included in the meta-analysis. Merging the effect sizes, we found no significant effect of folic acid supplementation on serum concentrations of IL-6 (WMD: −0.12, 95% CI −0.95 to 0.72 pg/mL, *p* = 0.780). Heterogeneity between studies was not significant (I^2^ = 47.5%, *p* = 0.149, Figure 3).

### 3.6. The Effect of Folic Acid Supplementation on Serum Concentrations of TNF-α

**Figure 4:**
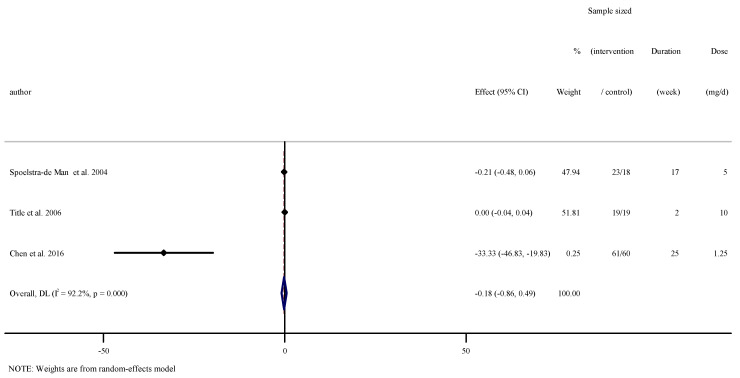
Forest plot detailing weighted mean difference and 95% confidence intervals (CIs) for the effect of folic acid supplementation on serum concentrations of TNF-α.

Overall results from three RCTs [24,33,39] including 200 participants did not reveal a significant effect of folic acid supplementation on serum concentrations of TNF-α (WMD: −0.18, 95% CI −0.86 to 0.49 pg/mL, *p* = 0.594). Significant between-study heterogeneity was observed (I^2^ = 92.2%, *p* < 0.001, Figure 4).

### 3.7. Sensitivity Analysis

We individually removed studies from the analysis to explore the impact of each study on the overall effect size. We did not find any significant effect of any separate study on the overall effect sizes of CRP, IL-6, and TNF-α.

### 3.8. Publication Bias

**Figure 5:**
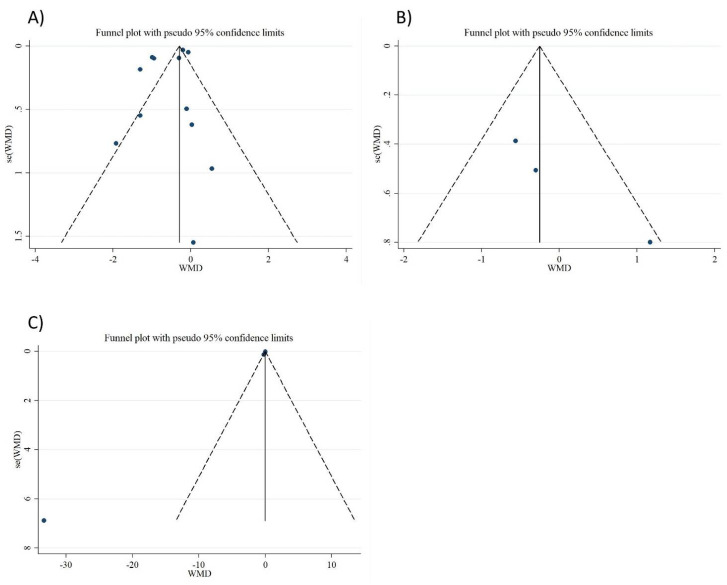
Funnel plot representing publication bias in the studies reporting the effect of folic acid on (**A**) CRP, (**B**) IL-6, and (**C**) TNF-α.

In the analysis of CRP, IL-6, and TNF-α, the funnel plot and Egger’s tests did not show any significant publication bias (*p* = 0.178, *p* = 0.127, *p* = 0.255, respectively, Figure 5A–C).

### 3.9. Meta-Regression and Non-Linear Dose–Response Analysis

**Figure 6:**
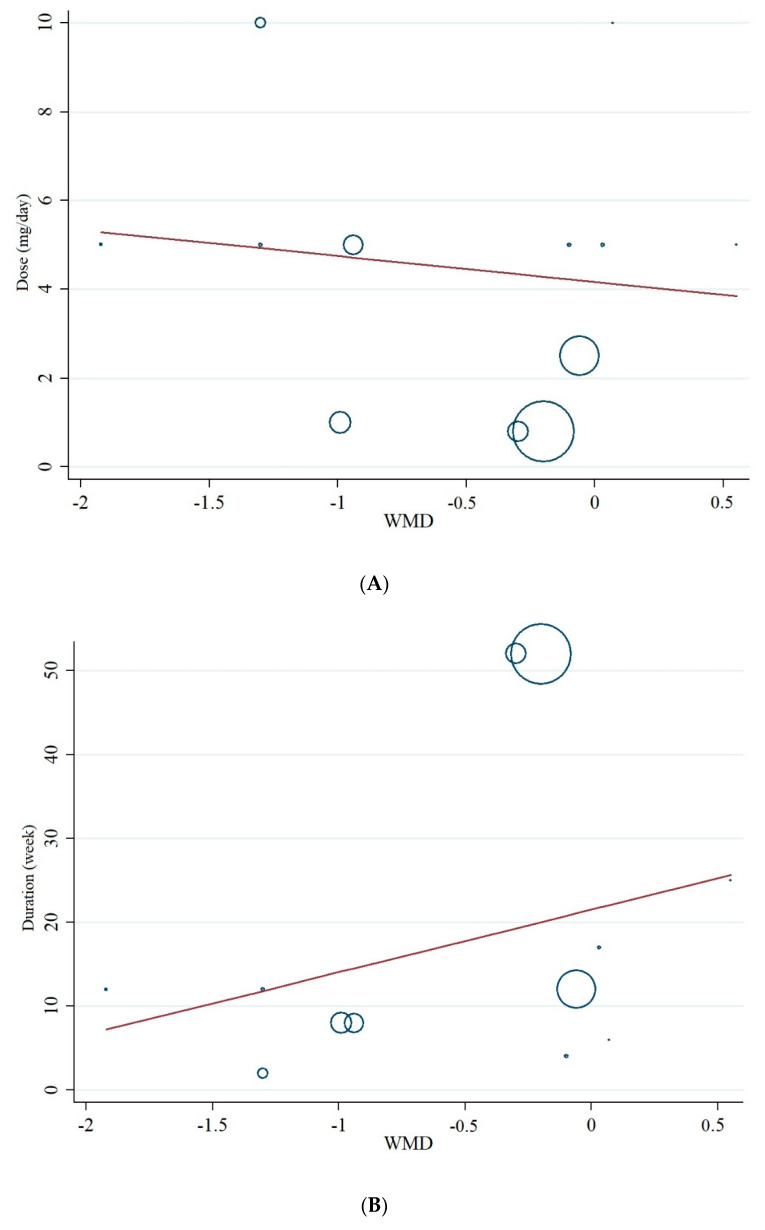
Linear meta-regression plots of the association between (**A**) dosage of folic acid and (**B**) duration of intervention and weighted mean difference of serum concentrations of CRP.

**Figure 7:**
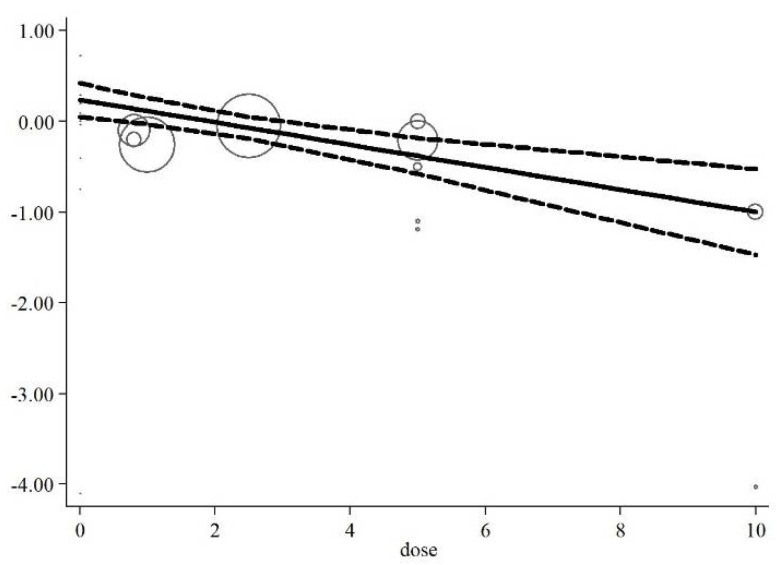
Non-linear dose–response of the association between dosage of folic acid supplementation and weighted mean difference of serum concentrations of CRP.

Meta-regression analysis did not indicate a linear relationship between dosage (*p* = 0.668) and duration of the intervention (*p* = 0.316) with changes in serum concentrations of CRP (*p* = 0.413, Figure 6A,B). In addition, the non-linear dose–response analysis showed a significant relationship between an elevated dosage of folic acid supplementation on decreasing CRP concentrations (*p* = 0.002, Figure 7).

## 4. Discussion

We investigated the effects of folic acid supplementation on inflammatory markers. Our findings indicated that folic acid supplementation decreases serum concentrations of CRP. In addition, we observed that folic acid supplementation significantly attenuated serum concentrations of CRP in all subgroups (baseline CRP, duration of intervention, supplement dosage, participants’ age and gender). However, we found no significant effect on serum concentrations of IL-6 and TNF-α.

CRP is an acute-phase protein that is often employed in clinical practice as an indicator of inflammation. This acute-phase protein is stimulated by several cytokines, including TNF-α, IL-6, and interleukin 1 beta (IL-1β), and is rapidly produced by the liver [42,43]. This protein also indicates low-grade inflammation in chronic diseases such as infections [44,45]. It has been previously established that CRP is often affected by the individual’s nutritional status, including folic acid deficiencies and other vitamins [46,47,48]. In line with our study, a previous meta-analysis by Fatahi et al. suggested that folic acid supplementation could significantly lower serum concentrations of CRP [49]. We expanded on this prior meta-analytic work by including further studies, performing a GRADE assessment as well as a meta-regression and linear and non-linear dose–response analyses, which enhances the quality of our findings. Moreover, several observational investigations have reported that folic acid concentrations are negatively related to CRP concentrations in various cohorts [42,46,50]. In the studies included in the current meta-analysis, Bahmani et al. showed that folic acid supplementation (5 mg/d) for 12 weeks in participants with polycystic ovary syndrome (PCOS) could reduce serum concentrations of CRP [37]. On the contrary, Asemi et al. [38] showed that long-term (six months) folic acid supplementation (5 mg/d) did not influence serum concentrations of CRP in patients with grade 1 cervical intraepithelial neoplasia. Furthermore, no significant influence on plasma concentrations of CRP was observed in participants with atherosclerosis after 400 μg/d of folic acid supplementation for 12 weeks [51]. The discrepancies among these findings may be explained by the different designs and methodologies used in previous studies, including different populations, intervention durations, supplement dosage, bioavailability, and purity of folic acid supplements.

The exact mechanisms underlying the influences of folic acid supplementation on inflammatory markers remain unclear. However, we propose some potential mechanisms for the correlation between folic acid supplementation and inflammatory markers in our meta-analysis. Reports have shown that homocysteine (Hcy) stimulates the expression of inflammatory cytokines, possibly by elevating poly adenosine diphosphate (ADP) ribose polymerase activation and prompting nuclear factor kappa B (NF-kB) activation. Since the Hcy-lowering influences of folic acid supplementation have been well documented [40,52], this supplement may result in the reduction of inflammatory markers by decreasing the activity of NF-kB [40,53] and the reduced poly ADP ribose polymerase activation [54]. Another mechanism may involve a folic-acid-induced amelioration of the hypoxia-stimulated inflammatory cytokines of human monocytic cells through inhibition of the phosphoinositide 3-kinases (PI3K)/protein kinase B (Akt)/hypoxia-inducible factor 1-alpha (HIF-1α) pathway [55].

Moreover, this vitamin has anti-inflammatory properties on TNF-α, lipopolysaccharide (LPS)-induced nitric oxide (NO), IL-1β via repression of NF-κB, and mitogen-activated protein kinase (MAPK) activation in RAW 264.7 macrophages [56]. These mechanisms may be described as notable influences of folic acid supplementation on serum concentrations of CRP. Although decreases in serum concentrations of CRP and the attenuating NF-κB pathway can result in IL-6 and TNF-α decrements [57], the lower number of included studies for IL-6 and TNF-α might explain the inconsistent results of folic acid supplementation on serum concentrations of these markers. Finally, it has been previously shown that oxidative stress is linked with lipid peroxidation and the development of inflammation [15,58,59]. Due to its potential antioxidant properties [60], folic acid supplementation may suppress lipid peroxidation and oxidative stress, initiating the metabolism and decrease of inflammation indirectly [61,62]. Further mechanistic studies are needed to elucidate the physiological processes involved in improving CRP following folic acid supplementation.

It is well-established that folic acid fortification and supplementation are safe [63]. According to the Institute of Medicine (IOM) guidelines, the tolerable upper intake level (UL) of folic acid (but not total food folate) is 1000 micrograms (1 mg) of folic acid, only because of possible neurological damage of vitamin B12 deficiency at levels exceeding 1000 micrograms [64]. The daily mentioned dosage of folic acid in included studies in our analysis varied between 0.8 and 10 mg/d. Although included studies did not report any side effects such as neurological damage, high doses of folic acid supplementation (more than 1 mg/d) should be consumed with caution.

Nevertheless, these outcomes should be interpreted with caution and some limitations should be borne in mind. We did not investigate the influences of folic acid supplementation on oxidative stress factors, other inflammatory markers, or plasma concentrations of folate, which would have helped to explain some of our findings. There was also a high heterogeneity among investigations. Moreover, the sample size for evaluating serum concentrations of IL-6 and TNF-α was smaller than that of CRP (222 and 200 vs. 1279, respectively); therefore, the current study had lower power to detect differences for these inflammatory cytokines. Future large-scale investigations are needed to evaluate the impact of folic acid supplementation on IL-6 and TNF-α.

In conclusion, the present systematic review and meta-analysis of RCTs demonstrated that folic acid supplementation decreases serum concentrations of CRP. Moreover, we found reduced serum concentrations of CRP in all subgroups (baseline CRP, duration of intervention, supplement dosage, participants’ age and gender). However, folic acid supplementation did not affect serum concentrations of IL-6 and TNF-α. Future investigations are needed to confirm and expand on our outcomes.

## Figures and Tables

**Table 2:** Characteristics of included studies in the meta-analysis.

Studies	Country	Study Design	Participant	Sample Size and Sex	Sample Size		Trial Duration (Week)	Means Age		Means BMI		Intervention		
					IG	CG		IG	CG	IG	CG	Acid Folic Dose (mg/d)	Control Group	Adverse Effects
Mangoni et al., 2003 [23]	United Kingdom	RA/PC (parallel)	healthy smokers	24: 9M, 15F	12	12	4	39.7 ± 11.77	36 ± 12.47	25.7 ± 2.77	24.9 ± 3.11	5	Placebo	No
Spoelstra-de Man et al., 2004 [33]	The Netherlands	RA/DB/PC (parallel)	type 2 diabetes mellitus	41: 24M, 10F	23	18	17	63.7 ± 8.6	66.1 ± 8.5	29.3 ± 3.9	28.8 ± 3.4	5	Placebo	NR
Durga et al., 2005 [34]	The Netherlands	RA/DB/PC (parallel)	men and postmenopausal women	530: 378M, 152F	264	266	52	60 ± 5	60 ± 6	NR	NR	0.8	Placebo	NR
Klerk et al., 2005 [35]	The Netherlands	RA/DB/PC (parallel)	older adults	276	137	139	52	59.5 ± 5.8	60.2 ± 5.2	26.8 ± 3.9	26.8 ± 3.9	0.8	Placebo	NR
Solini et al., 2006 [41]	Italy	RA/PC (parallel)	overweight subjects	60: 19M, 41F	30	30	12	50 ± 7	49 ± 8	27.5 ± 0.6	27.4 ± 0.6	2.5	Placebo	NR
Title et al., 2006 [24]	Canada	RA/DB/PC (cross-over)	type 2 diabetes	19: 9M,10F	19	19	2	54.5 ± 5.9	54.5 ± 5.9	NR	NR	10	Placebo	NR
Moens et al., 2007 [36]	Belgium	RA/DB/PC (cross-over)	acute myocardial infarction	40: 35M, 5F	20	20	6	57 ± 11	56 ± 14	NR	NR	10	Placebo	No
Bahmani et al., 2014 (A) [37]	Iran	RA/DB/PC (parallel)	overweight and obese women with polycystic ovary syndrome	46: 46F	23	23	8	24.1 ± 5.4	24.9 ± 5.9	26.1 ± 6.2	27.6 ± 5.7	1	Placebo	NR
Bahmani et al., 2014 (B) [37]	Iran	RA/DB/PC (parallel)	overweight and obese women with polycystic ovary syndrome	46: 46F	23	23	8	25.1 ± 4.9	24.9 ± 5.9	29 ± 5.9	27.6 ± 5.7	5	Placebo	NR
Asemi et al., 2016 [38]	Iran	RA/DB/PC (parallel)	cervical intraepithelial neoplasia grade 1	58: 58F	29	29	25	36.8 ± 8.8	39.1 ± 9.1	28.2 ± 3.5	29.8 ± 6.4	5	Placebo	No
Chen et al., 2016 [39]	China	RA/SB (parallel)	Alzheimer’s disease	121: 61M, 60F	61	60	25	68.1 ± 8.5	67.63 ± 7.92	23.25 ± 3.06	23.58 ± 4.28	1.25	No intervention	NR
Talari et al. 2016 [22]	Iran	RA/DB/PC (parallel)	metabolic syndrome	60: 26M, 34F	30	30	12	62.1 ± 9.6	65.4 ± 11.5	29.8 ± 3.8	29.8 ± 4.4	5	Placebo	NR
Bahmani et al., 2018 [35]	Iran	RA/DB/PC (parallel)	endometrial hyperplasia	60: 60F	30	30	12	44.4 ± 6.5	44.7 ± 3.1	30.7 ± 4.6	30.5 ± 3.8	5	Placebo	No

Abbreviations: IG, intervention group; CG, control group; DB, double-blinded; SB, single-blinded; PC, placebo-controlled; CO, controlled; RA, randomized; NR, not reported; F, female; M, male; NR, not reported.

**Table 3:** Subgroup analyses of folic acid supplementation on inflammation.

	NO	WMD (95%CI)	*P*-value	Heterogeneity			
				P Heterogeneity	I^2^	P between Sub-Groups	Tau-Squared
Subgroup analyses of folic acid supplementation on serum concentraitons of CRP							
Overall effect	12	−0.59 (−0.85, −0.32)	<0.001	<0.001	91.3%		0.1186
Baseline CRP (mg/L)							
<3	7	−0.44 (−0.73, −0.14)	0.003	<0.001	95.4%	<0.001	0.1186
≥3	5	−1.19 (−1.72, −0.65)	<0.001	0.284	20.5%		0.0907
Trial duration (week)							
≤8	5	−0.99 (−1.19, −0.79)	<0.001	0.152	40.4%	<0.001	0.0242
>8	7	−0.20 (−0.36, −0.05)	0.008	0.007	65.9%		0.0157
Intervention dose (mg/d)							
<5	4	−0.37 (−0.68, −0.07)	0.014	<0.001	96.4%	<0.001	0.0611
≥5	8	−0.89 (−1.27, −0.51)	<0.001	0.057	49.0%		0.1139
Age (year)							
<50	5	−0.92 (−1.13, −0.71)	<0.001	0.203	32.7%	<0.001	0.0558
>50	7	−0.40 (−0.64, −0.16)	0.001	<0.001	87.8%		0.0238
Sex							
Both sexes	8	−0.38 (−0.62, −0.15)	0.001	<0.001	85.8%	<0.001	0.0541
Male	4	−0.96 (−1.09, −0.83)	<0.001	0.397	0.0%		0.0000
Health status							
Healthy	4	−0.16 (−0.27, −0.06)	0.002	0.057	60.2%		0.006
Type 2 diabetes	2	−0.76 (−2.04, 0.51)	0.239	0.040	76.2%	<0.001	0.674
Polycystic ovary syndrome	2	−0.96 (−1.13, −0.79)	<0.001	0.775	0.0%		0.0000
Subgroup analyses of folic acid supplementation on serum concentraitons on IL-6							
Overall effect	3	−0.11 (−0.95, 0.71)	0.780	0.149	47.5%		0.2572
Subgroup analyses of folic acid supplementation on serum concentraitons of TNF-α							
Overall effect	3	−0.18 (−0.85, 0.49)	0.594	<0.001	92.2%		0.2278

Abbreviations: CI, confidence interval; WMD, weighted mean differences; CRP, c-reactive protein; IL-6, interleuline 6; TNF-α, tumor necrosis factor α.

## References

[1] Stankov S.V. (2012). Definition of inflammation, causes of inflammation and possible anti-inflammatory strategies. Open Inflamm. J..

[2] Suzuki K. (2019). Chronic inflammation as an immunological abnormality and effectiveness of exercise. Biomolecules.

[3] Ashtary-Larky D., Lamuchi-Deli N., Milajerdi A., Bakhtiar Salehi M., Alipour M., Kooti W., Ashtary-Larky P., Alamiri F., Sheikhi A., Afrisham R. (2017). Inflammatory and biochemical biomarkers in response to high intensity resistance training in trained and untrained men. Asian J. Sports Med..

[4] Zatterale F., Longo M., Naderi J., Raciti G.A., Desiderio A., Miele C., Beguinot F. (2020). Chronic adipose tissue inflammation linking obesity to insulin resistance and type 2 diabetes. Front. Physiol..

[5] Soysal P., Arik F., Smith L., Jackson S.E., Isik A.T. (2020). Inflammation, Frailty and Cardiovascular Disease. Adv. Exp. Med. Biol..

[6] Zhang Y., Anoopkumar-Dukie S., Arora D., Davey A.K. (2020). Review of the anti-inflammatory effect of SIRT1 and SIRT2 modulators on neurodegenerative diseases. Eur. J. Pharmacol..

[7] Moosavian S.P., Paknahad Z., Habibagahi Z., Maracy M. (2020). The effects of garlic (*Allium sativum*) supplementation on inflammatory biomarkers, fatigue, and clinical symptoms in patients with active rheumatoid arthritis: A randomized, double-blind, placebo-controlled trial. Phytother. Res..

[8] Murata M. (2018). Inflammation and cancer. Environ. Health Prev. Med..

[9] Esser N., Paquot N., Scheen A.J. (2015). Anti-inflammatory agents to treat or prevent type 2 diabetes, metabolic syndrome and cardiovascular disease. Expert Opin. Investig. Drugs.

[10] Kalmarzi R.N., Naleini S.N., Ashtary-Larky D., Peluso I., Jouybari L., Rafi A., Ghorat F., Heidari N., Sharifian F., Mardaneh J. (2019). Anti-Inflammatory and Immunomodulatory Effects of Barberry (Berberis vulgaris) and Its Main Compounds. Oxidative Med. Cell. Longev..

[11] Bagheri R., Rashidlamir A., Ashtary-Larky D., Wong A., Alipour M., Motevalli M.S., Chebbi A., Laher I., Zouhal H. (2020). Does green tea extract enhance the anti-inflammatory effects of exercise on fat loss?. Br. J. Clin. Pharmacol..

[12] Moosavian S.P., Rahimlou M., Saneei P., Esmaillzadeh A. (2020). Effects of dairy products consumption on inflammatory biomarkers among adults: A systematic review and meta-analysis of randomized controlled trials. Nutr. Metab. Cardiovasc. Dis..

[13] Galland L.J. (2010). Diet and inflammation. Nutr. Clin. Prac..

[14] Gunter B.R., Butler K.A., Wallace R.L., Smith S.M., Harirforoosh S. (2016). Non-steroidal anti-inflammatory drug-induced cardiovascular adverse events: A meta-analysis. J. Clin. Pharm. Ther..

[15] Asbaghi O., Ashtary-Larky D., Bagheri R., Nazarian B., Olyaei H.P., Kelishadi M.R., Nordvall M., Wong A., Dutheil F., Naeini A.A. (2021). Beneficial effects of folic acid supplementation on lipid markers in adults: A GRADE-assessed systematic review and dose-response meta-analysis of data from 21,787 participants in 34 randomized controlled trials. Crit. Rev. Food Sci. Nutr..

[16] Asbaghi O., Naeini F., Ashtary-Larky D., Moradi S., Zakeri N., Eslampour E., Kelishadi M.R., Naeini A.A. (2021). Effects of chromium supplementation on lipid profile in patients with type 2 diabetes: A systematic review and dose-response meta-analysis of randomized controlled trials. J. Trace Elem. Med. Biol..

[17] Asbaghi O., Fatemeh N., Mahnaz R.K., Ehsan G., Elham E., Behzad N., Damoon A.-L., Amirmansour A.N. (2020). Effects of chromium supplementation on glycemic control in patients with type 2 diabetes: A systematic review and meta-analysis of randomized controlled trials. Pharmacol. Res..

[18] Stanhewicz A.E., Kenney W.L. (2017). Role of folic acid in nitric oxide bioavailability and vascular endothelial function. Nutr. Rev..

[19] Mönch S., Netzel M., Netzel G., Ott U., Frank T., Rychlik M. (2014). Folate bioavailability from foods rich in folates assessed in a short term human study using stable isotope dilution assays. Food Funct..

[20] Qin X., Huo Y., Xie D., Hou F., Xu X., Wang X. (2013). Homocysteine-lowering therapy with folic acid is effective in cardiovascular disease prevention in patients with kidney disease: A meta-analysis of randomized controlled trials. Clin. Nutr..

[21] Pieroth R., Paver S., Day S., Lammersfeld C. (2018). Folate and Its Impact on Cancer Risk. Curr. Nutr. Rep..

[22] Talari H., Rafiee M., Farrokhian A., Raygan F., Bahmani F., Mofrad M.D., Hamidian Y., Tamtaji O.R., Karamali F., Asemi Z. (2016). The Effects of Folate Supplementation on Carotid Intima-Media Thickness and Metabolic Status in Patients with Metabolic Syndrome. Ann. Nutr. Metab..

[23] Mangoni A.A., Arya R., Ford E., Asonganyi B., Sherwood R.A., Ouldred E., Swift C.G., Jackson S.H. (2003). Effects of folic acid supplementation on inflammatory and thrombogenic markers in chronic smokers. A randomised controlled trial. Thromb. Res..

[24] Title L.M., Ur E., Giddens K., McQueen M.J., Nassar B.A. (2006). Folic acid improves endothelial dysfunction in type 2 diabetes–An effect independent of homocysteine-lowering. Vasc. Med..

[25] Gariballa S., Afandi B., AbuHaltem M., Yassin J., Habib H., Ibrahim W. (2013). Oxidative damage and inflammation in obese diabetic Emirati subjects supplemented with antioxidants and B-vitamins: A randomized placebo-controlled trail. Nutr. Metab..

[26] Moher D., Liberati A., Tetzlaff J., Altman D.G. (2009). Preferred reporting items for systematic reviews and meta-analyses: The PRISMA statement. Ann. Intern. Med..

[27] Higgins J.P.T., Altman D.G., Gøtzsche P.C., Jüni P., Moher D., Oxman A.D., Savović J., Schulz K.F., Weeks L., Sterne J.A.C. (2011). The Cochrane Collaboration’s tool for assessing risk of bias in randomised trials. BMJ.

[28] Borenstein M., Hedges L.V., Higgins J.P.T., Rothstein H.R. (2009). Introduction to Meta-Analysis.

[29] DerSimonian R., Kacker R. (2007). Random-effects model for meta-analysis of clinical trials: An update. Contemp. Clin. Trials.

[30] Sahebkar A. (2013). Are Curcuminoids Effective C-Reactive protein-lowering agents in clinical practice? Evidence from a meta-Analysis. Phytother. Res..

[31] Xu C., Doi S.A.R. (2018). The robust error meta-regression method for dose–response meta-analysis. JBI Evid. Implement..

[32] Gordon H., Oxman A., Vist G., Kunz R., Falck-Ytter Y., Alonso-Coello P., Schünemann H.J. (2008). Rating quality of evidence and strength of recommendations: GRADE: An emerging consensus on rating quality of evidence and strength of recommendations. BMJ.

[33] Spoelstra-de Man A., Brouwer C., Terheggen F., Bollen J., Stehouwer C., Smulders Y. (2004). No effect of folic acid on markers of endothelial dysfunction or inflammation in patients with type 2 diabetes mellitus and mild hyperhomocysteinaemia. Medicine.

[34] Durga J., Van Tits L., Schouten E.G., Kok F.J., Verhoef P. (2005). Effect of lowering of homocysteine levels on inflammatory markers: A randomized controlled trial. Arch. Intern. Med..

[35] Klerk M., Durga J., Schouten E.G., Kluft C., Kok F.J., Verhoef P. (2005). No effect of folic acid supplementation in the course of 1 year on haemostasis markers and C-reactive protein in older adults. Thromb. Haemost..

[36] Moens A.L., Claeys M.J., Wuyts F.L., Goovaerts I., Van Hertbruggen E., Wendelen L.C., Van Hoof V.O., Vrints C.J. (2007). Effect of folic acid on endothelial function following acute myocardial infarction. Am. J. Cardiol..

[37] Bahmani F., Karamali M., Shakeri H., Asemi Z. (2014). The effects of folate supplementation on inflammatory factors and biomarkers of oxidative stress in overweight and obese women with polycystic ovary syndrome: A randomized, double-blind, placebo-controlled clinical trial. Clin. Endocrinol..

[38] Asemi Z., Vahedpoor Z., Jamilian M., Bahmani F., Esmaillzadeh A. (2016). Effects of long-term folate supplementation on metabolic status and regression of cervical intraepithelial neoplasia: A randomized, double-blind, placebo-controlled trial. Nutrients.

[39] Chen H., Liu S., Ji L., Wu T., Ji Y., Zhou Y., Zheng M., Zhang M., Xu W., Huang G. (2016). Folic acid supplementation mitigates Alzheimer’s disease by reducing inflammation: A randomized controlled trial. Mediat. Inflamm..

[40] Bahmani F., Galougahi F.R., Vahedpoor Z., Jamilian M., Mahmoodi S., Baghban R., Bagherian T., Mehrizi M.Z., Asemi Z. (2018). The Effects of Folic Acid Supplementation on Recurrence and Metabolic Status in Endometrial Hyperplasia: A Randomized, Double-Blind, Placebo-Controlled Trial. Arch. Iran. Med..

[41] Solini A., Santini E., Ferrannini E. (2006). Effect of short-term folic acid supplementation on insulin sensitivity and inflammatory markers in overweight subjects. Int. J. Obes..

[42] González-Fernández D., Pons E.D.C., Rueda D., Sinisterra O.T., Murillo E., Scott M.E., Koski K.G. (2017). C-reactive protein is differentially modulated by co-existing infections, vitamin deficiencies and maternal factors in pregnant and lactating indigenous Panamanian women. Infect. Dis. Poverty.

[43] Ghafourian M., Ashtary-Larky D., Chinipardaz R., Eskandary N., Mehavaran M. (2016). Inflammatory Biomarkers’ Response to Two Different Intensities of a Single Bout Exercise Among Soccer Players. Iran. Red Crescent Med. J..

[44] Sullivan Z., Wong E.B., Ndung’U T., Kasprowicz V.O., Bishai W.R. (2015). Latent and active tuberculosis infection increase immune activation in individuals co-infected with HIV. EBioMedicine.

[45] De Souza A.B., Okawa R.T., Silva C.O., Araújo M.G. (2017). Short-term changes on C-reactive protein (CRP) levels after non-surgical periodontal treatment in systemically healthy individuals. Clin. Oral Investig..

[46] Kim H., Hwang J.-Y., Ha E.-H., Park H., Ha M., Lee S.-J., Hong Y.-C., Chang N. (2010). Association of maternal folate nutrition and serum C-reactive protein concentrations with gestational age at delivery. Eur. J. Clin. Nutr..

[47] Silva M.C., Furlanetto T.W. (2015). Does serum 25-hydroxyvitamin D decrease during acute-phase response? A systematic review. Nutr. Res..

[48] Gebreselassie S.G., Gase F.E., Deressa M.U. (2013). Prevalence and correlates of prenatal vitamin A deficiency in rural Sidama, Southern Ethiopia. J. Health Popul. Nutr..

[49] Fatahi S., Pezeshki M., Mousavi S., Teymouri A., Rahmani J., Varkaneh H.K., Ghaedi E. (2019). Effects of folic acid supplementation on C-reactive protein: A systematic review and meta-analysis of randomized controlled trials. Nutr. Metab. Cardiovasc. Dis..

[50] Haynes B.M.H., Pfeiffer C.M., Sternberg M.R., Schleicher R.L. (2013). Selected physiologic variables are weakly to moderately associated with 29 biomarkers of diet and nutrition, NHANES 2003–2006. J. Nutr..

[51] Mierzecki A., Kłoda K., Jastrzębska M., Chełstowski K., Honczarenko K., Kozłowska-Wojciechowska M., Naruszewicz M. (2012). Is there an effect of folic acid supplementation on the coagulation factors and C-reactive protein concentrations in subjects with atherosclerosis risk factors?. Postępy Hig. Med. Doświadczalnej.

[52] Brönstrup A., Hages M., Prinz-Langenohl R., Pietrzik K. (1998). Effects of folic acid and combinations of folic acid and vitamin B-12 on plasma homocysteine concentrations in healthy, young women. Am. J. Clin. Nutr..

[53] Papatheodorou L., Weiss N. (2007). Vascular Oxidant Stress and Inflammation in Hyperhomocysteinemia. Antioxid. Redox Signal..

[54] Xie J.-J., Yu X., Liao Y.-H., Chen J., Yao R., Chen Y., Liao M.Y., Ding Y., Tang T.T., Cheng X. (2009). Poly(ADP-Ribose) polymerase inhibition attenuates atherosclerotic plaque development in ApoE^−/−^ mice with hyperhomocysteinemia. J. Atheroscler. Thromb..

[55] Huang X., He Z., Jiang X., Hou M., Tang Z., Zhen X., Liang Y., Ma J. (2016). Folic acid represses hypoxia-induced inflammation in THP-1 cells through inhibition of the PI3K/Akt/HIF-1α pathway. PLoS ONE.

[56] Feng D., Zhou Y., Xia M., Ma J. (2011). Folic acid inhibits lipopolysaccharide-induced inflammatory response in RAW264.7 macrophages by suppressing MAPKs and NF-κB activation. Inflamm. Res..

[57] Tak P.P., Firestein G.S. (2001). NF-κB: A key role in inflammatory diseases. J. Clin. Investig..

[58] Niki E. (2008). Lipid peroxidation products as oxidative stress biomarkers. BioFactors.

[59] Suzuki K., Tominaga T., Ruhee R.T., Ma S. (2020). Characterization and modulation of systemic inflammatory response to exhaustive exercise in relation to oxidative stress. Antioxidants.

[60] Joshi R., Adhikari S., Patro B., Chattopadhyay S., Mukherjee T. (2001). Free radical scavenging behavior of folic acid: Evidence for possible antioxidant activity. Free Radic. Biol. Med..

[61] Kostyuk V.A., Potapovich A.I., Suhan T.O., de Luca C., Korkina L.G. (2011). Antioxidant and signal modulation properties of plant polyphenols in controlling vascular inflammation. Eur. J. Pharmacol..

[62] Asbaghi O., Ghanavati M., Ashtary-Larky D., Bagheri R., RezaeiKelishadi M., Nazarian B., Nordvall M., Wong A., Dutheil F., Suzuki K. (2021). Effects of Folic Acid Supplementation on Oxidative Stress Markers: A Systematic Review and Meta-Analysis of Randomized Controlled Trials. Antioxidants.

[63] Field M., Stover P.J. (2017). Safety of folic acid. Ann. N. Y. Acad. Sci..

[64] Finglas P.M., Allen L., Bailey L. (2000). Dietary Reference intakes for thiamin, riboflavin, niacin, vitamin B6, folate, vitamin B12, pantothenic acid, biotin and choline. Trends Food Sci. Technol..

